# A Cost-Utility Analysis of Lung Cancer Screening and the Additional Benefits of Incorporating Smoking Cessation Interventions

**DOI:** 10.1371/journal.pone.0071379

**Published:** 2013-08-07

**Authors:** Andrea C. Villanti, Yiding Jiang, David B. Abrams, Bruce S. Pyenson

**Affiliations:** 1 The Schroeder Institute for Tobacco Research and Policy Studies, Legacy, Washington, D. C., United States of America; 2 Department of Health, Behavior and Society, Johns Hopkins Bloomberg School of Public Health, Baltimore, Maryland, United States of America; 3 Milliman, Incorporated, New York, New York, United States of America; 4 Department of Oncology, Georgetown University Medical Center and Lombardi Comprehensive Cancer Center, Washington, D. C., United States of America; The University of Texas M. D. Anderson Cancer Center, United States of America

## Abstract

**Background:**

A 2011 report from the National Lung Screening Trial indicates that three annual low-dose computed tomography (LDCT) screenings for lung cancer reduced lung cancer mortality by 20% compared to chest X-ray among older individuals at high risk for lung cancer. Discussion has shifted from clinical proof to financial feasibility. The goal of this study was to determine whether LDCT screening for lung cancer in a commercially-insured population (aged 50–64) at high risk for lung cancer is cost-effective and to quantify the additional benefits of incorporating smoking cessation interventions in a lung cancer screening program.

**Methods and Findings:**

The current study builds upon a previous simulation model to estimate the cost-utility of annual, repeated LDCT screenings over 15 years in a high risk hypothetical cohort of 18 million adults between age 50 and 64 with 30+ pack-years of smoking history. In the base case, the lung cancer screening intervention cost $27.8 billion over 15 years and yielded 985,284 quality-adjusted life years (QALYs) gained for a cost-utility ratio of $28,240 per QALY gained. Adding smoking cessation to these annual screenings resulted in increases in both the costs and QALYs saved, reflected in cost-utility ratios ranging from $16,198 per QALY gained to $23,185 per QALY gained. Annual LDCT lung cancer screening in this high risk population remained cost-effective across all sensitivity analyses.

**Conclusions:**

The findings of this study indicate that repeat annual lung cancer screening in a high risk cohort of adults aged 50–64 is highly cost-effective. Offering smoking cessation interventions with the annual screening program improved the cost-effectiveness of lung cancer screening between 20% and 45%. The cost-utility ratios estimated in this study were in line with other accepted cancer screening interventions and support inclusion of annual LDCT screening for lung cancer in a high risk population in clinical recommendations.

## Introduction

Despite reductions in cigarette consumption and adult smoking prevalence in the years following publication of the 1964 Surgeon General's Report [1], [2] and aggressive tobacco control interventions over the past twenty years [3], lung cancer has remained the leading cause of cancer death among men in the United States since the mid-1950s and among women, since the late 1980s [4]. Lung cancer survival is directly linked to the stage at diagnosis, with five-year probability of survival of 52% for localized disease, 24% for regional disease, and 4% for disease distant metastases [4]. Few cases (15%) of lung cancer are diagnosed at the localized stage when survival is best [4].

This study is the third in a series that applied actuarial mortality and payer cost analytics to the feasibility of early lung cancer detection and treatment. The first study explored the huge mortality differences between early stage and late stage lung cancer that emerge from national cancer registry data [5] and concluded that the difference could not be explained by well-known testing biases, such as lead-time bias [6]. The second study examined the ability of repeated annual low dose CT (LDCT) screening to detect cancer at an earlier stage in a high risk population using a mortality and screening and treatment cost model for the commercially-insured population [7]. This paper demonstrated low payer costs for LDCT screening for lung cancer in per member per month (PMPM) terms: $0.76 PMPM for 2012 dollars compared to $2.50, $1.10, and $0.95 PMPM for Breast, Cervical and Colorectal cancer screening, respectively (2006 dollars). This second study reported that repeated LDCT screenings resulted in a low cost per life-year saved below $19,000 in the base case and below $27,000 (2012 USD) in the highest cost scenario, which is lower than current dollar estimates for cervical or breast cancer screening methods currently recommended by the U.S. Preventive Services Task Force [7].

Since our first study was published, a large randomized controlled trial, the National Lung Screening Trial (NLST), was stopped early when data showed a 20% reduction in lung cancer mortality in the LDCT screening arm compared to the chest x-ray (CXR) arm after a baseline and two follow-up scans, with no significant adverse effects of the screening program [8]. A recent commentary noted that the 20% mortality reduction seen in the NLST is inherently an underestimate of the lung cancer case-fatality rate and thus, the true mortality reduction is likely greater than 20% [9]. In 2011, the NLST study group published results for the trial in individuals (aged 55–74) at high risk for lung cancer due to smoking history of at least 30 pack-years [10]. LDCT identified a greater proportion of individuals with local disease (stage IA or IB) compared to CXR (63.0% vs. 47.6%) and a lower proportion of individuals with distant disease (stage IIIB or IV; 20.5% vs. 30.5%) [10]. The NLST's use of now-dated screening technology, community standard treatment, and only three annual screenings suggests a much higher mortality reduction through current, more precise LDCT technology combined with best practice treatment standards and extension of repeated screening beyond the NLST's three annual screens.

Despite the large population-based studies and randomized controlled trials of LDCT screening for lung cancer, there continues to be debate on recommending screening at the population level. A 2012 systematic review of LDCT screening for lung cancer by Bach et al. concluded that screening may benefit individuals at high risk of lung cancer, though the bulk of the conclusions focused on the potential harms of screening due to follow-up investigations, biopsies, and surgical procedures in patients with benign lesions [11]. This review formed the basis for clinical recommendations recommending annual LDCT lung cancer screening in a high risk population - those aged 55–74 with a smoking history of 30 pack-years or more who either currently smoke or have quit within the past 15 years [12]. Subsequently, errors in the review discovered by the authors and others have raised concerns about Bach et al.'s conclusions [13].

Given the tremendous potential for improved diagnosis, treatment and survival from lung cancer screening, several studies have been conducted to examine the cost-effectiveness of these programs. Two studies from 2001 simulated the effect of LDCT compared to no screening in a hypothetical cohort aged 60–74 at high risk of lung cancer over a five-year time horizon; both used data from the Early Lung Cancer Action Project (ELCAP) cohort study [14] to inform the distribution of stage at diagnosis in the LDCT group and also used the Surveillance, Epidemiology and End Results (SEER) program [5] for stage at diagnosis in the no screening group [15], [16]. The first study reported the incremental cost-effectiveness of a one-time screening at $15,274 (1999 USD; $25,064 in 2012 USD) per life year saved in a high prevalence scenario and up to $58,183 (1999 USD; $95,478 in 2012 USD) per life year saved assuming low lung cancer prevalence when accounting for a lead-time bias of one year [15]. The second study extended the model to assess the impact of repeat annual screenings on cost-effectiveness over five years and demonstrated incremental cost-effectiveness of $61,723 (1999 USD; $101,287 in 2012 USD) per life year saved and $50,473 (1999 USD; $82,826 in 2012 USD) per quality-adjusted life year saved accounting for one-year decrease in the survival benefit [16]. The conclusion of both papers was that LDCT screening for lung cancer appears to be cost-effective in high risk, elderly populations. Two other simulation modeling studies from the U.S. and Australia argue that LDCT screening for lung cancer is unlikely to be cost-effective [17], [18]. A 2003 simulation modeling study by Mahadevia et al. estimating the incremental cost-effectiveness of annual helical CT screening compared to no screening in hypothetical cohorts of current, quitting and heavy former smokers aged 60 years showed that annual helical CT screening could reach cost-effectiveness if favorable estimates for influential parameters were used simultaneously and argued that this screening modality was unlikely to be cost-effective in a heavy smoking population [17]. Similarly, a 2005 Australian study of LDCT screening for lung cancer in a hypothetical cohort of current male smokers aged 60 and above reported that this intervention was unlikely to be cost-effective assuming society's willingness to pay $50,000 per life year saved unless it achieved greater than a 20% reduction in lung cancer mortality [18]. These two studies' negative findings depend on lower effectiveness of screening than demonstrated by the NLST. McMahon et al.'s results from a patient-level microsimulation study, again using lower effectiveness of screening than NLST, showed that annual screening of current and former smokers aged 50 to 74 years would cost between $154,000 and $207,000 (2012 USD) per quality-adjusted life year saved, compared to no screening intervention and assuming background quit rates among current smokers [19]. In this study, lung cancer-specific mortality was reduced by 18% to 25% at 10-year follow-up in the hypothetical cohorts of persons with at least 20 pack-years of smoking history who received smoking cessation counseling and annual CT screenings for lung cancer.

An important distinction in comparing lung cancer screening to other cancer screening is the concentration of lung cancer risk among former or current smokers. Among adults with lung cancer, 21% report being current smokers, 61% former smokers and 18% never smokers [20]. By contrast, mammography is recommended for all women within certain ages and colorectal cancer screening is recommended for all men and women within certain ages. This risk concentration for lung cancer reduces the size of the population needing screening and also coincides with focused smoking cessation opportunities. Several studies have identified lung cancer screening as a “teachable moment” to improve smoking cessation in this heavy smoking population [21]–[23], possibly via changes to risk perceptions among current and former smokers [24]. A study from the Early Lung Cancer Action Project showed that 23% of active smokers reported quitting after a baseline CT scan [25], a more than four-fold increase over the background quit rate in the general population of approximately 4% [26]. A recent modeling study bases its estimate of the cost-effectiveness of CT lung cancer screening programs solely on the inclusion of smoking cessation outcomes of its participants [19]. The incorporation of smoking cessation counseling and treatment in lung cancer screening is likely to achieve greater savings in medical costs and reductions in morbidity and mortality than screening alone. It is also likely to appeal to employers and commercial payers given recent estimates of the excess annual healthcare costs of a smoking employee to a private employer [27].

The current study builds upon our two previous studies [6], [7] and our actuarial model [7] to estimate the cost per quality-adjusted life year (QALY) saved through LDCT screening, and shows the impact of integrating various smoking cessation programs for screened, current smokers using a commercial payer perspective. The goal of this study is to determine the cost effectiveness of LDCT screening for lung cancer in a hypothetical population of adults aged 50–64 at high risk for lung cancer and to quantify the additional benefits of incorporating smoking cessation interventions in a lung cancer screening program.

## Methods

Details on the previous model have been published elsewhere [7]; briefly, the model adopted a commercial payer (actuarial) perspective and quantified costs and effects of lung cancer screening and associated smoking cessation interventions over a 15-year time horizon. Screening costs were established using U.S. Medicare-reimbursement fees (assuming no patient cost sharing) to both screening and follow-up of suspicious nodules, most of which were not cancer. We set the year of analysis in 2012 and assumed all current smokers and half of the former smokers between age 50 and 64 to be eligible for lung cancer screening, with eligibility set as at least 30 pack-years of smoking history. A pack-year is defined as the equivalent of smoking one pack of cigarettes per day for one year. Using data from the 2010 National Health Interview Survey on cigarette smoking status for those aged 45–64 [28], this resulted in approximately 30% of the US population being eligible for lung cancer screening. In our hypothetical cohort, two-thirds of individuals eligible for screening are current smokers and one-third are former smokers. Cancer treatment costs were determined from a large payer database, Truven Marketscan, and included all hospital, physician, ancillary and drug costs eligible for insurer reimbursement. To allow use of actual insurance program cost information available in large commercial claims databases for the costs of cancer treatment, clinical stages IA and IB were modeled as stage A, clinical stages IIA, IIB, and IIIA were modeled as stage B, and clinical stages IIIB and IV as stage C, using a previous published algorithm [7]. The A, B, C stages correspond approximately to SEER's localized, regional, distant categories [29]. All care costs were tabulated, without any attempt to isolate costs not associated with cancer or to attribute any non-medical costs such as lost productivity, and average population medical costs by age and gender were applied to persons without cancer. The model calculated expected future life years through the use of survival probabilities for each age, gender and lung cancer stage (or no lung cancer). All costs were converted to 2012 dollars. The model's outputs were the incremental costs (screening and treatment) and quality-adjusted life years saved during the model period and future life-years after the model period comparing 100% screening to 0% screening.

The current study expands the previous model by estimating the QALYs saved by lung cancer screening and treatment, incorporating the impact of smoking cessation interventions on costs, health care costs saved, and QALYs saved, and addressing the impact of lung cancer screening on economic output. As with the earlier model, a 2-year lead time was assumed for all screening-detected cancer and we used the New York ELCAP data to inform our base case. In this model, we estimate the incremental effect of screening or screening plus cessation treatment over no screening. Table 1 presents the input parameters of the model described in this section.

**Table 1:** Input parameters for lung cancer screening model.

Parameter	Base case estimate	Sensitivity analyses	Reference
**Costs**			
Lung cancer screening			
Average annual cost of low dose spiral CT screening for lung cancer	$210	125%, 150%	[7]
Lung cancer treatment in first year of diagnosis			
Average cost, Stage A	$82,087	n/a	[7]
Average cost, Stage B	$132,464	n/a	[7]
Average cost, Stage C	$142,750	n/a	[7]
Smoking cessation treatment			
Average cost per smoking cessation counseling session	$83	n/a	Thomson Reuters Marketscan 2010 trended to 2012
Average cost of generic nicotine replacement therapy per quit attempt	$228	n/a	Thomson Reuters Marketscan 2010 trended to 2012
Average cost of generic bupropion per quit attempt	$290	n/a	Thomson Reuters Marketscan 2010 trended to 2012
Average cost of varenicline (Chantix) per quit attempt	$379	n/a	Thomson Reuters Marketscan 2010 trended to 2012
Average health care costs incurred per quit	$0	$12,031, $12,093	Base case: [33]–[35] Sensitivity estimates for light, intensive cessation programs
**Survival probabilities**	**%**		
Estimated annual survival			
Male, current smoker	45.7		[32]
Male, ex-smoker	63.0	+/−5%	[32]
Male, never smoker	68.0		[32]
Female, current smoker	64.6		[32]
Female, ex-smoker	69.6	+/−5%	[32]
Female, never smoker	82.8		[32]
Lung cancer survival			
Male, Stage A	90.4–96.6		[6]
Male, Stage B	71.8–89.9		[6]
Male, Stage C	26.3–73.7		[6]
Female, Stage A	92.1–97.4		[6]
Female, Stage B	72.6–90.9		[6]
Female, Stage C	27.4–76.0		[6]
**Screening probabilities**			
Baseline LDCT scan			
Negative result (no nodules present or semi-positive)	79.0		[30], personal communication with C. Henschke
*Positive result (nodule>5 mm)*	*21.0*		[30], personal communication with C. Henschke
*Follow-up scan in 3 months given positive result*	*19.0*		[30], personal communication with C. Henschke
Repeat scan at 1 year given follow-up scan	99.0		[30], personal communication with C. Henschke
Biopsy given follow-up scan	1.0		[30], personal communication with C. Henschke
Surgery given biopsy after follow-up scan	90.0		[30], personal communication with C. Henschke
*Biopsy given positive result*	*2.0*		[30], personal communication with C. Henschke
Repeat scan at 1 year given biopsy	76.0		[30], personal communication with C. Henschke
Surgery given biopsy	24.0		[30], personal communication with C. Henschke
Repeat LDCT scan (annual)			
Negative result (no nodules present or semi-positive)	93.0		[30], personal communication with C. Henschke
*Positive result (nodule>5 mm)*	*7.0*		[30], personal communication with C. Henschke
*Antibiotics and follow-up scan within 1 month given positive result*	*3.0*		[30], personal communication with C. Henschke
Repeat scan at 1 year given antibiotics and follow-up scan	5.0		[30], personal communication with C. Henschke
Additional follow-up scan at 3 months given antibiotics and follow-up scan	95.0		[30], personal communication with C. Henschke
Biopsy given additional follow-up scan	5.0		[30], personal communication with C. Henschke
Surgery given biopsy after antibiotics and follow-up scan	90.0		[30], personal communication with C. Henschke
Biopsy given antibiotics and follow-up scan	1.0		[30], personal communication with C. Henschke
Surgery given biopsy after antibiotics and follow-up scan	90.0		[30], personal communication with C. Henschke
*Follow-up scan within 6 months given positive result*	*4.0*		[30], personal communication with C. Henschke
Repeat scan at 1 year given follow-up scan	99.0		[30], personal communication with C. Henschke
Biopsy given follow-up scan	1.0		[30], personal communication with C. Henschke
Surgery given biopsy after follow-up scan	90.0		[30], personal communication with C. Henschke
**Lung cancer probabilities**	**%**	**%**	
Status quo			
Stage A	17.4		[5]
Stage B	14.6		[5]
Stage C	68.0		[5]
Lung cancer screening	New York ELCAP	NLST	
Stage A	79.3	63.0	[10], [30]
Stage B	16.2	16.5	[10], [30]
Stage C	4.5	20.5	[10], [30]
**Smoking probabilities, %**	**%**		
Male, aged 18+, current smoker	21.2		[28]
Male, aged 18+, ex-smoker	25.5		[28]
Female, aged 18+, current smoker	17.5		[28]
Female, aged 18+, ex-smoker	17.3		[28]
Probability of participation in smoking cessation treatment among current smokers	19.2	+/−10%	Calculated from [28]
Annual reduction in smoking prevalence with increasing age (Background quit rate)	2.5		Calculated from [28]
**Effectiveness of smoking cessation intervention at 12 months**	**Odds ratio**		
Light cessation intervention (behavioral treatment only)	1.5 (midpoint of 1.3–1.7)		[37]
Pharmacological treatment only	2.55 (midpoint of 1.5–3.6)		[37]
Combined behavioral and pharmacological treatment, over either intervention alone	1.5 (midpoint of 1.3–1.7)		[37]
Intensive cessation intervention (combined behavioral and pharmacological treatment)	3.04		Calculated from [37]
**Quality-adjusted life years**	**Utility weight**		
Utility for general population, by age			
Males, aged 50–59	0.819	+/−10%	[33]
Males, aged 60–69	0.803	+/−10%	[33]
Males, aged 70–79	0.770	+/−10%	[33]
Males, aged 80–89	0.742	+/−10%	[33]
Females, aged 50–59	0.788	+/−10%	[33]
Females, aged 60–69	0.784	+/−10%	[33]
Females, aged 70–79	0.748	+/−10%	[33]
Females, aged 80–89	0.700	+/−10%	[33]
Utility weights for lung cancer patients			
Stage A	0.823	+/−10%	[34]
Stage B	0.772	+/−10%	[34]
Stage C	0.573	+/−10%	[34]
QALYs saved by smoking cessation			
Males, aged 55–64	2.25		[38]
Females, aged 55–64	2.01		[38]

Unless stated, all costs are presented in 2012 dollars.

### Cost of lung cancer screening, treatment and smoking cessation programs

We used a previous published [7] estimate of the cost of annual lung cancer screening, which was developed by applying Medicare reimbursement to follow-up decision tree logic published for a large observational study. Costs included all follow-up from suspicious nodules identified in screening; in the first year of screening, 21% of participants required follow-up LDCT scan or biopsy for a positive result (nodules larger than five millimeters in diameter) and in subsequent years, positive results from screening dropped to 7% (see Exhibit 1 and the Appendix in [7] for detailed decision trees). These values were based on the New York ELCAP [30] and personal communication with the ELCAP lead investigator (Claudia Henschke, December 6, 2010). They are slightly higher than those reported in the New York ELCAP (14% at baseline and 6% at follow-up) [30] and I-ELCAP studies (16% at baseline) [31] and slightly lower than the 27% at baseline reported by the NLST, though NLST defined a positive result as greater than four, rather than five, millimeters in diameter [10]. The original model included one brief anti-smoking counseling session for each person screened, which was priced using a 2012 Medicare reimbursement rate. In the current modeling, we included the cost of alternative types of cessation programs only for smokers and used a typical, commercial reimbursement rate for these programs. We used previously published estimates for the cost of lung cancer treatment for stages A, B and C, which were developed from a large claims database of commercially-insured people. Our model is retrospective in that it assumes that screening started 15 years prior. We used 2012 cost levels throughout our work instead of applying cost levels of prior years, and we did not apply discount factors to account for the time value of money spent in years prior to 2012. Because medical cost inflation has greatly exceeded discount rates during the past 15 years, our 2012 cumulative tabulation would have produced lower costs had we used prior years' cost levels and multiplied by discount rates to bring costs to 2012 levels.

### Screening efficacy and stage shift

The previous model used data from the New York ELCAP for probability of detecting lung cancers at Stages A, B, and C using LDCT [30]. For this study, we show results for both the New York ELCAP data and recently published data on LDCT detection of lung cancer by stage in the NLST [10], which reported a lower portion of detected early stage lung cancers, presumably because it used only three annual screens and included older four-sensor LDCT equipment. We have not considered costs or effects associated with identified cancers that could be very slow growing or would resolve on their own in line with NLST's low estimate of such cases [10].

### Probability of survival

The hypothetical cohort in this study was comprised of individuals with a 30 pack-year history of smoking, and the probability of survival for those without lung cancer accounted for gender, age and smoking status (current or former), which were estimated using results from a 1997 paper by Schoenbaum [32]. For the patients with lung cancer, survival probabilities varied by age, gender, and lung cancer stage at diagnosis and were based on SEER [6]. We assume that current or former smokers with lung cancer have the same probability of survival.

### Quality-adjusted life years

Quality adjusted life years were estimated by multiplying the probability that an individual survives to each future year by a utility weight related to age, sex and stage of lung cancer. Utility weights for the general population of males and females aged 50–59 and 60–69 were obtained from a study using the SF-6D and standard gamble technique in a nationally-representative sample [33]. Among patients diagnosed with lung cancer, stage, age and sex-specific utilities were multiplied by the utility of lung cancer by stage as determined from a meta-analysis of lung cancer utility weights [34]. Patients with screen-detected cancers were assigned QALYs at ages that accounted for a two-year detection lead time. Lung cancer utilities were defined using the standard gamble method for non-metastatic non-small cell lung cancer (NSCLC), mixed/indeterminate NSCLC, and metastatic NSCLC, which we matched to stages A, B, and C, respectively. We multiply the age and sex dependent utility factors by the lung cancer stage specific utility factors. This reflected the lower quality-of-life at each of the three stages of lung cancer relative to the general quality-of-life by age and sex. For lung cancer patients who died during the analytic horizon (before reaching age 65), we assumed that they experienced the quality of life of a stage C lung cancer patient in their last three months of life. Summing the product of utility weights and annual survival probability across all modeled past and future years generated QALYs for the hypothetical cohort for the screening and non-screening scenarios. The difference in total past and future QALYs for the screening scenarios compared to the without screening scenario generated the incremental QALYs due to screening. Similar to costs, we did not discount QALYs saved by lung cancer screening, as we used a retrospective model. Additionally, when looking only at the impact of lung cancer screening, we assumed that smokers and former smokers had the same quality of life.

### Cost and Impact of Smoking Cessation Programs

In the previous model [7], costs for annual smoking cessation counseling were included in the lung cancer screening cost, but the effects of such counseling on life years and health care costs saved were not modeled. The current study models the costs and impact of no additional cessation program, a light program without pharmaceutical treatment, and three intensive programs each with a different pharmaceutical treatment, to be offered to all current smokers in conjunction with the annual screening. In the comparison group receiving no screening, we assume that persons quit at a specified background rate of 2.5% (calculated from [28]) and capture the incremental effect of cessation in the screening arm as the number of additional quits above the background rate. The modeled light cessation program consists of a single counseling session in addition to the LDCT screening. The intensive program involves up to four counseling sessions and 12 weeks of pharmaceutical treatment, as described in the 2008 Clinical Practice Guidelines on *Treating Tobacco Use and Dependence* [35]. These options were chosen to model a wide range of potential effects of smoking cessation intervention.

Input parameters on the costs and effectiveness of the smoking cessation interventions are presented in Table 1 and detailed methods are provided in a technical appendix (File S1). These estimates were derived from the 2010 National Health Interview Survey [28], [36], a large administrative claims database (Thomson Reuters Marketscan), a meta-analysis of cessation interventions [37], other cost-utility analyses of smoking cessation interventions [38]–[40], mortality rates in the general population [41] and in smokers [6], [32], and studies of the impact of quitting smoking on health care costs [42], [43]. QALYs saved by smoking cessation accounted for a 3% discount rate, a 3.5% background quit rate, and a 37% relapse rate [38].

### Sensitivity analyses

We conducted a range of sensitivity analyses to the test the robustness of our findings, including using NLST rather than ELCAP data on stage-shift as a result of LDCT screening for lung cancer, varying the utility weights used in estimating QALYs by ten percent, and increasing the cost of screening to 125% and 150%. We also present results for four types of smoking cessation interventions in two categories: light and intensive. Further, we examine the inclusion of health care costs incurred among quitters in the cost of the cessation program, as well as a ten percent change in the participation and quit rates of each program and the ex-smoker mortality rate. Because the medical cost component of the CPI tends to understate medical inflation we produced a sensitivity scenario by trending dollar values of other preventive health interventions to 2012 USD at twice the medical CPI.

## Results

Assuming annual LDCT screenings are given over 15 years to a cohort of high risk adults aged 50–64, the projected cost of lung cancer screening and treatment in the base case averaged $1.8 billion per year (totaling $27.8 billion) and yielded 985,284 QALYs over the 15-year period. The resulting cost-utility ratio comparing 100% participation in repeat annual LDCT screenings to no screening was $28,240 per QALY gained (Table 2). The light smoking cessation intervention consisting of behavioral treatment cost an additional $1.4 billion and saved an additional 273,566 QALYs. The intensive smoking cessation intervention consisted of combined behavioral and pharmacological treatment. In all scenarios, the QALYs saved by intensive cessation nearly doubled the QALYs saved by LDCT screening alone (930,754 QALYs). The additional costs of the intensive cessation intervention varied by medication: the generic NRT scenario cost $3.2 billion, the scenario using generic buproprion cost $4.1 billion and the cost of varenicline (Chantix) was $5.3 billion. Adding smoking cessation to these annual screenings resulted in increases in both the costs and QALYs saved, reflected in cost-utility ratios ranging from $16,198 (intensive intervention using generic NRT) to $23,185 (light intervention).

**Table 2:** Projected 15-year costs and quality-adjusted life years saved by lung cancer screening and treatment with and without smoking cessation using stage shifts from the NY-ELCAP and NLST in authors' actuarial model.

	NY-ELCAP stage shift	NLST stage shift
**Screening**		
Lung cancer screening and treatment costs	$27,824,282,242	$34,054,299,361
QALYs saved by screening and treatment	985,284	722,795
Cost per QALY saved	$28,240	$47,115
**Screening + light smoking cessation intervention**		
Additional costs for cessation	$1,361,556,665	$1,361,556,665
Additional QALYs saved by cessation	273,566	273,566
Cost per QALY saved	$23,185	$35,545
**Screening + intensive smoking cessation intervention**		
***A. NRT generic plus behavioral***		
Additional costs for cessation	$3,212,191,737	$3,212,191,737
Additional QALYs saved by cessation	930,754	930,754
Cost per QALY saved	$16,198	$22,537
***B. Bupropion generic plus behavioral***		
Additional costs for cessation	$4,088,822,965	$4,088,822,965
Additional QALYs saved by cessation	930,754	930,754
Cost per QALY saved	$16,656	$23,067
***C. Chantix plus behavioral***		
Additional costs for cessation	$5,342,861,783	$5,342,861,783
Additional QALYs saved by cessation	930,754	930,754
Cost per QALY saved	$17,310	$23,826

NY-ELCAP, New York Early Lung Cancer Action Project; NLST, National Lung Screening Trial; QALY, quality-adjusted life year.

Sensitivity analyses examined the robustness of the results to variations in model parameters. Table 2 presents estimates using NLST stage-shift data which resulted in a slightly higher cost-utility ratio of $47,115 for lung cancer screening alone and a range from $22,537 per QALY saved (intensive intervention using generic NRT) to $35,545 per QALY saved (light intervention) when adding a smoking cessation component to screening. One-way sensitivity analyses of the base case (Table 3) showed that lung cancer screening remained cost-effective to changes in the utility weights, a higher percentage of participants diagnosed in Stage A, and increased costs of LDCT screening. Sensitivity analyses of the cessation scenarios incorporated the health care costs incurred by those who quit over the 15-year period, 10% variation in the participation and quit rates of the cessation programs, and 5% variation in the mortality rate of former smokers. In all cases, lung cancer screening plus cessation remained highly cost-effective at less than $50,000 per QALY saved. Health care costs incurred over the 15-year period by quitting smoking through light cessation intervention were estimated at $1.5 billion and through the intensive cessation intervention at $5.3 billion. These costs equate to an average cost of $802 and $2,742 per quit attempt for the light and intensive cessation interventions, respectively, or $12,031 (light) and $12,093 (intensive) per successful quit.

**Table 3:** One-way sensitivity analysis of model parameters.

		Cost/QALY saved	
Parameter	Range	Lower limit	Upper limit
**Sensitivity analysis of the base case** a			
Utility weights	+/−10%	$22,367	$36,820
Participants diagnosed in Stage A	110%	$21,730	-
Cost of screening	125%, 150%	$36,421	$44,602
**Sensitivity analysis of the cessation scenarios**			
Health care costs incurred among quitters			
Light cessation intervention	$1,548,238,011	-	$24,414
Intensive cessation intervention^b^	$5,294,440,356	-	$20,073
Quit rate and participation rate in cessation program	+/−10%		
Light cessation intervention		$22,390	$23,969
Intensive cessation intervention^b^		$16,239	$18,487
Ex-smoker mortality rate^c^	+/−5%		
Light cessation intervention		$23,589	$25,293
Intensive cessation intervention^b^		$15,437	$22,042

a Base case uses data from the New York ELCAP study.

b For sensitivity analyses, the intensive cessation intervention consists of Chantix plus behavioral treatment.

c Accounts for health care costs incurred per quit as related to mortality.

### Other preventive health interventions

We compared the cost per QALY saved of the current LDCT screening protocol to studies of the cost-effectiveness of lung cancer screening and other preventive health interventions, including colon cancer screening [44], cervical cancer screening via Pap test [45], biennial mammography [46], type 2 diabetes screening [47], annual HIV testing [48], in-center dialysis [49], and cholesterol-lowering medication [50]. All costs have been trended to 2012 costs using the medical cost component of the CPI. Costs calculated in foreign currency were first converted into U.S. dollars for that year, updated to account for per capita health expenditures in that country compared to the U.S., and then trended using the CPI. Table 4 presents the comparison of results from our model to previously-published cost-utility analyses of other preventive health interventions. Colonoscopy every ten years and annual fecal occult blood screening for colorectal cancer in adults aged 50–75 were highly cost-effective interventions, as was cervical cancer screening in women aged 20–65 every three years with the Papanicolaou (Pap) test. The estimated cost-utility of annual LDCT screening for lung cancer was in line with Pap test for cervical cancer and superior to biennial mammography screening for breast cancer. Lung cancer screening was more cost-effective than type 2 diabetes screening in adults, annual HIV testing in a high risk population, in-center dialysis for end stage renal disease, and cholesterol-lowering medication.

**Table 4:** Comparison of lung cancer screening with LDCT to other preventive health interventions.

Intervention	Original value	Year	$/QALY saved (2012 USD)	$/QALY saved (2012 USD, sensitivity analysis)	Consistent with USPSTF guidelines	Reference
**Lung cancer screening with LDCT in high risk population**						
Annual screening over 15 years, aged 50–64	$28,240–$47,115	2012 USD	$28,240–$47,115	-	Under review	
**Other preventive health interventions**						
Colonoscopy every 10 years, ages 50–75	$4,870	2008 CAN	$8,552	$9,625	Yes	[44]
Annual fecal occult blood screening for colorectal cancer, ages 50–75	$15,991–$18,595	2008 CAN	$28,080–$32,652$31,604–$36,750	Yes	[44]	
Papanicolaou (Pap) test for cervical cancer, every 3 years in women aged 20–65	$11,835	2000 USD	$18,662	$28,940	Yes	[45]
Biennial mammography and clinical breast exam in women, aged 50–75 years	$34,000	2000 USD	$53,611	$83,139	Yes	[46]
Type 2 diabetes screening, ages 25+	$56,649	1995 USD	$105,650	$192,741	No	[47]
Annual HIV testing in high risk population	$100,000	2001 USD	$150,745	$223,909	Yes	[48]
In-center dialysis vs. no renal replacement therapy	$129,200	2000 USD	$203,724	$315,928	Yes	[49]
Cholesterol-lowering medication (statin) vs. Step I diet^a^	$130,000–$260,000	1997 USD	$227,878–$455,755	$391,442–$782,883	-	[50]

USD, U.S. dollars; CAN, Canadian dollars.

a Among men with LDL> = 160 mg/dL.

### Economic impact of lung cancer screening

By reducing deaths of individuals during productive years, lung cancer screening is also likely to increase economic output. To provide insight into this issue, we estimated the incremental annual wages due to screening, and the resulting taxes and total economic impact associated with those wages for people under age 65, assuming the current portion of workers by age and gender [51]. Over the fifteen-year period, we estimated $4.8 billion in wages gained, $1.7 billion in income taxes gained, and $10.6 billion in GDP added. Excluding all costs and effects related to smoking cessation, this would mean that for every dollar spent on lung cancer screening, society would recover $0.38 of its investment. If included in our calculation, this would further reduce the cost per QALY saved.

## Discussion

Building on our two other studies [6], [7], this simulation study finds that repeat annual lung cancer screenings in a high risk cohort of adults aged 50–64 is highly cost-effective at $28,240 per QALY gained compared to both the currently accepted cost-effectiveness threshold of $109,000 per QALY gained [52], [53] and the more conservative threshold of $50,000 per QALY gained. The cost-utility of our lung cancer screening protocol is comparable to colorectal or cervical cancer screening and superior to breast cancer screening which are all are recommended by U.S. Preventive Services Task Force (USPSTF). Lung cancer screening is less costly than other recommended interventions like HIV testing in a high risk population or in-center dialysis for end-stage renal disease.

The range of costs per QALY saved in our study ($28,240–$47,115) is approximately 50% lower than some previous estimates [17], [18] of the cost-effectiveness of lung cancer screening with LDCT. These other studies used assumptions that LDCT was significantly less effective at reducing mortality or detecting early stage lung cancer than demonstrated by either ELCAP or NLST. Another difference between the current study and previous models is our use of a younger cohort with a lower incidence of lung cancer; by diagnosing fewer cases of lung cancer, we would expect our model to yield a similar or less favorable cost-utility ratio compared to those using an older hypothetical cohort.

Even at older ages, the risk of lung cancer mortality among current smokers is substantially reduced by smoking cessation [54]. Linking smoking cessation interventions with the annual screening program improved the cost-effectiveness of lung cancer screening between 20% and 45% by increasing the number of QALYs saved. These findings were robust to inclusion of the additional health care costs incurred by quitters living longer than continuing smokers, as well as variation in participation in the cessation interventions, successful cessation, and mortality among quitters.

Our findings emphasize the unprecedented public health potential of lung cancer screening as a medical home for smokers and ex-smokers to motivate cessation and other health behavior change. For example, the addition of coronary artery calcium scores (CACS) measured by LDCT scan to an existing risk prediction model has been shown to improve risk classifications for coronary heart disease [55]. Estimating CACS and grading emphysema, chronic obstructive pulmonary disease, and lung damage on annual scans could promote personalized medicine and maximize teachable moments. While concerns about radiation exposure associated with the increased use of diagnostic imaging studies seem likely to encourage thoughtful use [56], the average effective dose of radiation from annual LDCT screening for lung cancer [57] is likely to be roughly equivalent to exposure from biennial mammography screening for breast cancer [58] over the long term. The utility of a single tool (LDCT screening) to identify risk and promote behavior change across multiple disease processes represents a cost-saving paradigm in preventive health care that could help to reduce the $96 billion in annual medical costs attributable to smoking [59] and have a tremendous benefit in a population at high risk for the top three leading causes of death in the US.

Our model likely overestimates the cost per QALY saved by lung cancer screening in several ways. First, we used 2012 cost levels throughout our work. Our cost projection is a retrospective model, in that we examine results for 2012 if screening had started 15 years ago. Since medical inflation has been much higher than either general inflation or risk free interest (financial discount) rates, had we applied a present value calculation to lower historical costs, our cost per QALY saved would be significantly lower. From a forecast perspective, our assumption is consistent with the view that healthcare spending maintains its current, high portion of GDP and does not further increase or decline. Similarly, estimates of the QALYs gained per quit in our model used a 3% discount rate [38]. Since we adopted a conservative, commercial payer perspective and assumed medical inflation to equal the discount rate, we have not discounted costs. We note that non-discounted costs are a standard practice for health cost projections by the U.S. Congressional Budget Office as well as in the Property-Casualty insurance industry in the U.S. Based on relationships in the paper by Javitz et al., we estimate that not discounting QALYs would reduce the cost per QALY saved through cessation by roughly 50%. Third, our application of SEER mortality to stages A, B and C likely understates the mortality advantages of screen-detected cancers. We used the historical distributions of the traditional stages Ia, Ib, IIa, IIB, IIIa, IIIb and IV when we mapped SEER mortality to stages A, B, and C. However, it seems likely that screen detected cancers will be more heavily weighted toward the earlier traditional stages within each of our categories A, B, and C, especially because of the progress in LDCT scans detecting smaller nodules, which would increase the live-years saved. We also used a higher rate of positive results than reported by the New York ELCAP or I-ELCAP studies at baseline and follow-up screens, which likely overestimates the follow-up costs for false positive results over the study period. Finally, our cumulative cohort methodology captures 15 annual screenings at age 50 but only one screening for age 64. Because the lung cancer incidence increases with age, our approach overweights the ages with the highest cost/benefit ratio relative to a steady state of screening.

Our sensitivity analyses included increasing the price of lung cancer screening by 50% of our estimated cost and using stage-shift data from both the I-ELCAP and NLST studies. Lung cancer screening remained cost-effective in all scenarios. NLST shows a smaller stage shift than the New York ELCAP figures we utilized in our base case and resulted in a lower mortality reduction. The smaller stage shift can be understood by the limits of NLST—only three annual screens and inclusion of older, four-slice LDCT technology. By default, NLST used community standard practice and not a protocol for follow-up (as used in the I-ELCAP), which may account for the lower observed mortality reduction. However, both I-ELCAP and NLST are voluntary programs and could reflect unknown biases. If future recommendations for lung cancer screening would include a protocol for follow-up, the base case of our cost-utility analysis using New York-ELCAP data may better reflect the effectiveness and cost-effectiveness of LDCT screening at the broader population level.

Results from our simulation model indicate that repeat annual LDCT lung cancer screening for adults aged 50–64 with 30+ pack-years of smoking history is highly cost-effective from a commercial payer perspective. Lung cancer screening becomes even more cost-effective when linked with smoking cessation interventions and this study presents cost-utility ratios across a range of programs consisting of a single counseling session up to a full course of combined behavioral and pharmacological treatment. Annual LDCT lung cancer screening in this high risk population remained cost-effective across all sensitivity analyses and we would expect this screening to become more favorable over time with increased identification of early stage cancers in the routine screening pool. The cost-utility ratios estimated in this study were in line with other cancer screening interventions endorsed by the USPSTF and support inclusion of annual LDCT screening for lung cancer in future USPSTF recommendations.

## Supporting Information

File S1 Cost and Impact of Smoking Cessation Programs. (DOCX)

## References

[1] Centers for Disease Control and Prevention (1999) Tobacco use–United States, 1900–1999. MMWR Morb Mortal Wkly Rep 48: 986–993. 10577492

[2] Centers for Disease Control and Prevention (2010) CDC grand rounds: current opportunities in tobacco control. MMWR Morb Mortal Wkly Rep 59: 487–492. 20431525

[3] Farrelly MC, Pechacek TF, Thomas KY, Nelson D (2008) The Impact of Tobacco Control Programs on Adult Smoking. Am J Public Health 98: 304–309. 18172148 10.2105/AJPH.2006.106377 PMC2376884

[4] American Cancer Society (2012) Cancer Facts & Figures 2012. Atlanta, GA: American Cancer Society.

[5] National Cancer Institute (2012) SEER Cancer Statistics Review 1975–2009: U.S. National Institutes of Health. Available: http://www.seer.cancer.gov/csr/1975_2009_pops09/index.html. Accessed June 1 2012.

[6] Goldberg SW, Mulshine JL, Hagstrom D, Pyenson BS (2010) An actuarial approach to comparing early stage and late stage lung cancer mortality and survival. Popul Health Manag 13: 33–46. 20158322 10.1089/pop.2009.0010

[7] Pyenson BS, Sander MS, Jiang Y, Kahn H, Mulshine JL (2012) An actuarial analysis shows that offering lung cancer screening as an insurance benefit would save lives at relatively low cost. Health Aff (Millwood) 31: 770–779. 22492894 10.1377/hlthaff.2011.0814

[8] Data Safety Monitoring Board, National Lung Screening Trial, National Cancer Institute (2010) Statement concerning the National Lung Screening Trial: National Cancer Institute. Available: http://www.cancer.gov/images/dsmb-nlst.pdf. Accessed May 6 2011.

[9] Yankelevitz DF, Smith JP (2013) Understanding the core result of the National Lung Screening Trial. N Engl J Med 368: 1460–1461. 10.1056/NEJMc1213744 23574139

[10] Aberle DR, Adams AM, Berg CD, Black WC, Clapp JD, et al (2011) Reduced lung-cancer mortality with low-dose computed tomographic screening. N Engl J Med 365: 395–409. 21714641 10.1056/NEJMoa1102873 PMC4356534

[11] Bach PB, Mirkin JN, Oliver TK, Azzoli CG, Berry DA, et al (2012) Benefits and Harms of CT Screening for Lung Cancer: A Systematic ReviewBenefits and Harms of CT Screening for Lung Cancer. JAMA 1–12. 10.1001/jama.2012.5521 PMC3709596 22610500

[12] National Comprehensive Cancer Network (2011) NCCN Clinical Practice Guidelines in Oncology: Lung Cancer Screening, Version 1.2012. Fort Washington, PA: National Comprehensive Cancer Network, Inc. Available: http://www.nccn.org/professionals/physician_gls/pdf/lung_screening.pdf. Accessed June 15 2012.

[13] Yip R, Islami F, Zhao S, Tao M, Yankelevitz DF, et al (2013) Errors in systematic reviews: an example of computed tomography screening for lung cancer. Eur J Cancer Prev 10.1097/CEJ.0b013e3283616290 23715405

[14] Henschke CI, McCauley DI, Yankelevitz DF, Naidich DP, McGuinness G, et al (1999) Early Lung Cancer Action Project: overall design and findings from baseline screening. Lancet 354: 99–105. 10408484 10.1016/S0140-6736(99)06093-6

[15] Marshall D, Simpson KN, Earle CC, Chu C (2001) Potential cost-effectiveness of one-time screening for lung cancer (LC) in a high risk cohort. Lung Cancer 32: 227–236. 11390004 10.1016/s0169-5002(00)00239-7

[16] Marshall D, Simpson KN, Earle CC, Chu CW (2001) Economic decision analysis model of screening for lung cancer. Eur J Cancer 37: 1759–1767. 11549429 10.1016/s0959-8049(01)00205-2

[17] Mahadevia PJ, Fleisher LA, Frick KD, Eng J, Goodman SN, et al (2003) Lung cancer screening with helical computed tomography in older adult smokers: a decision and cost-effectiveness analysis. JAMA 289: 313–322. 12525232 10.1001/jama.289.3.313

[18] Manser R, Dalton A, Carter R, Byrnes G, Elwood M, et al (2005) Cost-effectiveness analysis of screening for lung cancer with low dose spiral CT (computed tomography) in the Australian setting. Lung Cancer 48: 171–185. 15829317 10.1016/j.lungcan.2004.11.001

[19] McMahon PM, Kong CY, Bouzan C, Weinstein MC, Cipriano LE, et al (2011) Cost-effectiveness of computed tomography screening for lung cancer in the United States. J Thorac Oncol 6: 1841–1848. 21892105 10.1097/JTO.0b013e31822e59b3 PMC3202298

[20] Centers for Disease Control and Prevention (2007) Cigarette smoking among adults–United States, 2006. MMWR Morb Mortal Wkly Rep 56: 1157–1161. 17989644

[21] Cox LS, Clark MM, Jett JR, Patten CA, Schroeder DR, et al (2003) Change in smoking status after spiral chest computed tomography scan screening. Cancer 98: 2495–2501. 14635086 10.1002/cncr.11813

[22] Townsend CO, Clark MM, Jett JR, Patten CA, Schroeder DR, et al (2005) Relation between smoking cessation and receiving results from three annual spiral chest computed tomography scans for lung carcinoma screening. Cancer 103: 2154–2162. 15825210 10.1002/cncr.21045

[23] Taylor KL, Cox LS, Zincke N, Mehta L, McGuire C, et al (2007) Lung cancer screening as a teachable moment for smoking cessation. Lung Cancer 56: 125–134. 17196298 10.1016/j.lungcan.2006.11.015

[24] Park ER, Ostroff JS, Rakowski W, Gareen IF, Diefenbach MA, et al (2009) Risk perceptions among participants undergoing lung cancer screening: baseline results from the National Lung Screening Trial. Ann Behav Med 37: 268–279. 19711141 10.1007/s12160-009-9112-9 PMC2831282

[25] Ostroff JS, Buckshee N, Mancuso CA, Yankelevitz DF, Henschke CI (2001) Smoking cessation following CT screening for early detection of lung cancer. Prev Med 33: 613–621. 11716658 10.1006/pmed.2001.0935

[26] Burns D, Anderson C, Johnson M, Major J, Biener L, et al.. (2000) Cessation and cessation measures among adult daily smokers: national and state-specific data. Population-based smoking cessation: a conference on what works to influence smoking in the general population Smoking and Tobacco Control Monograph No 12. Bethesda, MD: National Cancer Institute.

[27] Berman M, Crane R, Seiber E, Munur M (2013) Estimating the cost of a smoking employee. Tob Control 10.1136/tobaccocontrol-2012-050888 23733918

[28] Schiller J, Lucas J, Ward B, Peregoy J (2012) Summary health statistics for U.S. adults: National Health Interview Survey, 2010. Vital Health Stat 10. 22834228

[29] National Cancer Institute Surveillance epidemiology and end results: SEER stat fact sheets: lung and bronchus. Bethesda, MD: NCI. Available: http://seer.cancer.gov/statfacts/html/lungb.html#survival. Accessed Mar 5 2012.

[30] New York Early Lung Cancer Action Project Investigators (2007) CT Screening for lung cancer: diagnoses resulting from the New York Early Lung Cancer Action Project. Radiology 243: 239–249. 17392256 10.1148/radiol.2431060467

[31] Henschke CI, Yip R, Yankelevitz DF, Smith JP (2013) International Early Lung Cancer Action Program I (2013) Definition of a positive test result in computed tomography screening for lung cancer: a cohort study. Ann Intern Med 158: 246–252. 23420233 10.7326/0003-4819-158-4-201302190-00004

[32] Schoenbaum M (1997) Do smokers understand the mortality effects of smoking? Evidence from the Health and Retirement Survey. Am J Public Health 87: 755–759. 9184501 10.2105/ajph.87.5.755 PMC1381045

[33] Hanmer J, Lawrence WF, Anderson JP, Kaplan RM, Fryback DG (2006) Report of nationally representative values for the noninstitutionalized US adult population for 7 health-related quality-of-life scores. Med Decis Making 26: 391–400. 16855127 10.1177/0272989X06290497

[34] Sturza J (2010) A review and meta-analysis of utility values for lung cancer. Med Decis Making 30: 685–693. 20448248 10.1177/0272989X10369004

[35] Tobacco Use and Dependence Guideline Panel (2008) Treating Tobacco Use and Dependence: 2008 Update. Rockville, MD: U.S. Department of Health and Human Services.

[36] Centers for Disease Control and Prevention (2011) Quitting smoking among adults — United States, 2001–2010. MMWR Morb Mortal Wkly Rep 60: 1513–1519. 22071589

[37] Abrams DB, Graham AL, Levy DT, Mabry PL, Orleans CT (2010) Boosting population quits through evidence-based cessation treatment and policy. Am J Prev Med 38: S351–363. 20176308 10.1016/j.amepre.2009.12.011 PMC4515751

[38] Javitz H, Swan G, Zbikowski S, Curry S, McAfee T, et al (2004) Cost-effectiveness of different combinations of bupropion SR dose and behavioral treatment for smoking cessation: a societal perspective. Am J Manag Care 10: 217–226. 15032259

[39] Fiscella K, Franks P (1996) Cost-effectiveness of the transdermal nicotine patch as an adjunct to physicians' smoking cessation counseling. JAMA 275: 1247–1251. 8601956

[40] Cromwell J, Bartosch WJ, Fiore MC, Hasselblad V, Baker T (1997) Cost-effectiveness of the clinical practice recommendations in the AHCPR guideline for smoking cessation. Agency for Health Care Policy and Research. JAMA 278: 1759–1766. 9388153

[41] Social Security Administration (2012) Actuarial Life Table: Period Life Table, 2007. Available: http://www.ssa.gov/oact/STATS/table4c6.html#ss. Accessed June 7 2012.

[42] Musich S, Faruzzi SD, Lu C, McDonald T, Hirschland D, et al (2003) Pattern of medical charges after quitting smoking among those with and without arthritis, allergies, or back pain. Am J Health Promot 18: 133–142. 14621408 10.4278/0890-1171-18.2.133

[43] Solberg LI, Maciosek MV, Edwards NM, Khanchandani HS, Goodman MJ (2006) Repeated tobacco-use screening and intervention in clinical practice - Health impact and cost effectiveness. Am J Prev Med 31: 62–71. 16777544 10.1016/j.amepre.2006.03.013

[44] Heitman SJ, Hilsden RJ, Au F, Dowden S, Manns BJ (2010) Colorectal cancer screening for average-risk North Americans: an economic evaluation. PLoS Med 7: e1000370. 21124887 10.1371/journal.pmed.1000370 PMC2990704

[45] Mandelblatt JS, Lawrence WF, Womack SM, Jacobson D, Yi B, et al (2002) Benefits and costs of using HPV testing to screen for cervical cancer. JAMA 287: 2372–2381. 11988058 10.1001/jama.287.18.2372

[46] Stout NK, Rosenberg MA, Trentham-Dietz A, Smith MA, Robinson SM, et al (2006) Retrospective cost-effectiveness analysis of screening mammography. J Natl Cancer Inst 98: 774–782. 16757702 10.1093/jnci/djj210

[47] CDC Diabetes Cost-Effectiveness Study Group (1998) The cost-effectiveness of screening for type 2 diabetes. CDC Diabetes Cost-Effectiveness Study Group, Centers for Disease Control and Prevention. JAMA 280: 1757–1763. 9842951

[48] Paltiel AD, Weinstein MC, Kimmel AD, Seage GR 3rd, Losina E, et al (2005) Expanded screening for HIV in the United States–an analysis of cost-effectiveness. N Engl J Med 352: 586–595. 15703423 10.1056/NEJMsa042088

[49] Winkelmayer WC, Weinstein MC, Mittleman MA, Glynn RJ, Pliskin JS (2002) Health economic evaluations: the special case of end-stage renal disease treatment. Med Decis Making 22: 417–430. 12365484 10.1177/027298902236927

[50] Prosser LA, Stinnett AA, Goldman PA, Williams LW, Hunink MG, et al (2000) Cost-effectiveness of cholesterol-lowering therapies according to selected patient characteristics. Ann Intern Med 132: 769–779. 10819699 10.7326/0003-4819-132-10-200005160-00002

[51] Bureau of Labor Statistics (2013) Labor Force Statistics from the Current Population Survey: A-3 Employment status of the civilian noninstitutional population by sex and age, seasonally adjusted: United States Department of Labor. Available: http://stats.bls.gov/web/empsit/cpseea03.htm. Accessed June 5 2013.

[52] Braithwaite RS, Meltzer DO, King JT Jr, Leslie D, Roberts MS (2008) What does the value of modern medicine say about the $50,000 per quality-adjusted life-year decision rule? Med Care 46: 349–356. 18362813 10.1097/MLR.0b013e31815c31a7

[53] Weinstein MC (2008) How much are Americans willing to pay for a quality-adjusted life year? Med Care 46: 343–345. 18362811 10.1097/MLR.0b013e31816a7144

[54] Doll R, Peto R, Boreham J, Sutherland I (2004) Mortality in relation to smoking: 50 years' observations on male British doctors. BMJ 328: 1519. 15213107 10.1136/bmj.38142.554479.AE PMC437139

[55] Polonsky TS, McClelland RL, Jorgensen NW, Bild DE, Burke GL, et al (2010) Coronary artery calcium score and risk classification for coronary heart disease prediction. JAMA 303: 1610–1616. 20424251 10.1001/jama.2010.461 PMC3033741

[56] Smith-Bindman R, Miglioretti DL, Johnson E, Lee C, Feigelson HS, et al (2012) Use of diagnostic imaging studies and associated radiation exposure for patients enrolled in large integrated health care systems, 1996–2010. JAMA 307: 2400–2409. 22692172 10.1001/jama.2012.5960 PMC3859870

[57] Aberle DR, Berg CD, Black WC, Church TR, Fagerstrom RM, et al (2011) The National Lung Screening Trial: overview and study design. Radiology 258: 243–253. 21045183 10.1148/radiol.10091808 PMC3009383

[58] Brenner DJ, Hall EJ (2007) Computed tomography–an increasing source of radiation exposure. N Engl J Med 357: 2277–2284. 18046031 10.1056/NEJMra072149

[59] Centers for Disease Control and Prevention (2008) Smoking-attributable mortality, years of potential life lost, and productivity losses–United States, 2000–2004. MMWR Morb Mortal Wkly Rep 57: 1226–1228. 19008791

