# Supplementary material for: A Cost-Utility Analysis of Lung Cancer Screening and the Additional Benefits of Incorporating Smoking Cessation Interventions
Source: PLoS One. 2013 Aug 7;8(8):e71379. doi: 10.1371/journal.pone.0071379 (PMC3737088; doi:10.1371/journal.pone.0071379)
Supplement: File S1 — (DOCX) [file pone.0071379.s001.docx]

**File S1. Cost and Impact of Smoking Cessation Programs**

Technical Appendix

In our model, two-thirds of individuals eligible for screening are current smokers, as all current smokers age 50-64 were eligible for screening, plus 50% more, to reflect quitters with 30-plus pack-years smoking history. We assume 45.5% of these current smokers will make a quit attempt in a given year, and based on recent data from the National Health Interview Survey (NHIS), of those who attempt to quit, 42.3% will take part in cessation programs.[36] Combining the two quit-related probabilities yields our estimate that 19.25% (45.5% x 42.3%) of at-risk smokers would begin participation in a cessation program, which we used for all cessation scenarios.

The modeled light cessation program consists of a single counseling session in addition to the LDCT screening. The intensive program involves up to four counseling sessions and 12 weeks of pharmaceutical treatment, as described in the 2008 Clinical Practice Guidelines on *Treating Tobacco Use and Dependence*.[35] We estimated the average commercial cost per counseling session at $83 without cost sharing, from our analysis of a large administrative claims database, Thomson Reuters Marketscan, which contains the claims experience for over 45 million commercially-insured lives. We assume that the alternative pharmaceutical treatments are nicotine replacement therapy, bupropion or varenicline (Chantix), and obtained from Thomson Reuters MarketScan 2010 (MarketScan 2010) the average allowed amount paid by commercial insurers for each alternative plus a dispensing fee of $1.60 per prescription. We trended the historical counseling costs at 1.9% per year based on the Bureau of Labor Statistics’ Consumer Price Index (CPI) for medical costs from 2010 to May 2012. We trended costs for generic nicotine replacement therapy (NRT), generic bupropion and varenicline costs to 2012 by 2%, 14% and 13% annually from 2010 to 2012 based on price data from Medispan, a large pharmaceutical market database. For individuals who begin intensive treatment, we assumed typical compliance obtained from a large commercial database, with 2 counseling sessions and 1.22, 2.85 and 1.37 30-day NRT, bupropion, and varenicline prescriptions, respectively. We estimated the cost of the intensive cessation program using NRT at $228, bupropion at $290 and varenicline at $379 per quit attempt in 2012.

Abrams et al[37] reported ranges of odds ratios for annual quit rates for the smoking cessation interventions relative to non-interventions. We used the midpoint of these ranges for each type of intervention. The light cessation intervention increases the odds of successful quitting by 50% (midpoint of 1.3 – 1.7) and the intensive cessation intervention increase the odds by a factor of 3.04, which accounts for combined behavioral and pharmacological treatment. Figures published in the NHIS 2010 imply a reduction of approximately 2.5% in smoking prevalence for each year of increasing age, with prevalence dropping from 21% for ages 50 to 64 down to 13% for ages 65 and 74.[28] The net quit rates over the intervention horizon of 15 years, incremental to the background quit rate, were 11.8% and 36.2% for the light and intensive programs, respectively. We assumed the same quit rate for the three alternative intensive programs with different pharmaceutical treatments.

We follow the participation of patients who were smokers at their first screening over time to estimate costs separately for the light and intensive smoking cessation interventions. In our model, in 2012, a screened patient age 50 has only one opportunity to participate in the cessation program, while someone at age 64 would have had 15 opportunities—once each year starting age 50.

The smoking cessation programs lead to increases in QALYs gained among quitters. We used estimates from the literature for QALYs gained by smoking cessation for males and females aged 55-64, which were 2.25 and 2.01 respectively.[38] These estimates assume a 3% discount rate, a 3.5% background quit rate, and a 37% relapse rate. The QALYs gained by lung cancer screening were modeled using utility weights.

We estimated the impact of quitting smoking on health care costs by using published ratios of costs of never smokers, current smoker and ex-smokers. The ratio of healthcare costs of never smokers to current and ex-smokers were report at 0.76 and 0.86, respectively.[42,43] According to 2010 estimates from the NHIS,[28] there are 21% current smokers, 25% ex-smokers and 53% never smokers. Using normative health care costs from 2009 Thomson Reuters MarketScan data, health care cost ratios by smoking status, and the portions of people in each smoking status, we estimated the annual healthcare cost for each age, gender and smoking status. To obtain the cumulative future total annual health cost, we multiplied the healthcare cost at future ages by the survival probability to that age, by gender and smoking status. We assumed that healthcare inflation equals discount rates, so applied 2012 cost levels throughout. The mortality rate for ex-smokers was not readily available, so we estimated the ex-smoker mortality rate by interpolating between the general population mortality rate[41] and the current smoker mortality rate.[6] The interpolation factors are based on the relativities of never, ex and current smoker survival probabilities to age 75 among whites aged 50-62[32] as shown in Table 1 and the proportion of the population in each of the three smoker categories from the 2010 NHIS data.[28]

In the base case, we assume that there is no change in lifetime medical costs incurred by quitting smoking per previous studies.[38-40] We calculate the additional costs for light and intensive smoking cessation programs by multiplying the future health costs incurred through quitting by the number of successful quits (above the background quit rate) through either program. We separately captured the increased costs due to quitters living longer than continuing smokers.

REFERENCES

See manuscript for corresponding references.
